# Construction and Optimization Strategy of an Ecological Network in Mountainous Areas: A Case Study in Southwestern Hubei Province, China

**DOI:** 10.3390/ijerph19159582

**Published:** 2022-08-04

**Authors:** Qian Zuo, Yong Zhou, Jingyi Liu

**Affiliations:** 1 Key Laboratory for Geographical Process Analysis & Simulation of Hubei Province, Central China Normal University, Wuhan 430079, China; 2 College of Urban and Environmental Sciences, Central China Normal University, Wuhan 430079, China

**Keywords:** ecological network, ecosystem services, morphological spatial pattern analysis, landscape connectivity, ecological function zones

## Abstract

High-intensity urban development and economic exploitation have led to the fragmentation and isolation of regional habitat patches, and biodiversity is under serious threat. Scientific identification and effective optimization of ecological networks are essential for maintaining and restoring regional ecosystem connectivity and guiding sustainable socio-economic development. Taking the mountainous areas of southwest Hubei Province (MASHP) in central China as an example, this study first developed a new integrated approach to identify ecological sources based on a quantitative assessment of ecosystem services and the morphological spatial pattern analysis (MSPA) method; it then used the Linkage Mapper tool to extract ecological corridors, applied the principle of hydrological analysis to identify ecological nodes, evaluated each ecological element to quantify its importance, and finally constructed the ecological network and further proposed some optimization countermeasures. The results show that the ecological network in the MASHP is dominated by ecological resources composed of forestland. Connectivity in the central region is significantly better than in other regions, including 49 ecological sources with an area of 3837.92 km^2^, 125 ecological corridors with a total length of 2014.61 km, and 46 ecological nodes. According to the spatial distribution of crucial ecological landscape elements, a complete and systematic ecological framework of “two verticals, three belts, three groups, and multiple nodes” was proposed. The internal optimization of the ecological network in mountainous areas should focus on improving ecological flow, and strategies such as enhancing the internal connectivity of ecosystems, unblocking ecological corridors, and dividing ecological functional zones can be adopted. Based on the above analyses, this study also made recommendations for ecological protection and development and construction planning in mountainous areas. This study can provide realistic paths and scientific guidelines for ecological security and high-quality development in the MASHP, and it can also have implications for the construction of ecological networks and comprehensive ecological management in other mountainous areas.

## 1. Introduction

The continuous socio-economic development and the increasing demand for urban construction land have resulted in the encroachment of a large amount of ecological land by construction land and the constant compression of ecological space, which has divided the originally continuous natural habitat into a mosaic of mixed patches with a high degree of fragmentation and has seriously affected the functioning of the ecosystem [1,2,3]. This has led to the destruction of ecological processes and serious degradation of the ecological environment, which to a certain extent has intensified the fragmentation of the regional landscape; meanwhile, the connectivity between habitat patches is decreasing, the habitat quality of species continues to decline, and the circulation between different ecological streams is hindered, all of which have posed serious challenges to the sustainable development of the region [4,5,6]. As a result, the concept of ecological security, which is based on maintaining the integrity, health, and sustainability of ecological processes, has gradually received widespread attention from governments and academics, and the theory has provided strong support for governments to seek a balance between regional ecological conservation and sustainable economic development [7,8]. After decades of development, ecological security has become one of the essential concepts to alleviate the contradiction between natural ecological conservation and human social evolution [9,10]. More and more scholars consider constructing an ecological network as a suitable method to solve the above thorny problems [11,12,13,14]. Ecological networks are derived from the theories and methods of landscape ecology and further enriched and developed on this basis. They specifically refer to a potential spatial pattern of the ecosystem to maintain the normal biodiversity, ecosystem health, and sustainable supply of ecosystem services in a specific region [15,16], which can characterize the integrity and health of the current natural ecosystem [17] and enhance the connectivity between different habitats through “point-line-surface”, to effectively resist the lasting adverse effects of habitat fragmentation on biodiversity [18,19].

At present, ecological network research is becoming more and more mature, and the mainstream paradigm of “sources identification-resistance surface construction-corridors extraction and nodes discrimination” has been formed [20,21,22]. As critical ecological patches, ecological sources can promote ecological processes and maintain the integrity and stability of the ecosystem [23], and they are identified mainly by the direct identification method, minimum area threshold method, and ecosystem service overlay analysis method. The first one is the direct identification method, which refers to the method of identifying specific areas of the research object, such as the green areas, forest parks, scenic spots and nature reserves with large concentrated and continuous areas and important recreational functions as ecological sources [24,25]. This method is simple and straightforward, but the screening criteria are relatively single and subjective. The second is the minimum area threshold method. For the ecological sources that have been roughly selected, data reduction is directly carried out according to previous experience, and the operation process is simplified. The choice of some thresholds remains controversial [26,27]. The third method is the ecosystem service overlay analysis, which evaluates the sensitivity and importance of ecosystems based on several important or typical ecosystem service capabilities, and uses them as the basis for identifying ecological sources. The method is widely used because of the comprehensive and relatively scientific nature of its quantitative indicators, but these ecosystem service functions are mainly related to human well-being and are not closely related to species migration and dispersal processes. Integrated approaches to identifying ecological sources have become a new research trend. The Integrated Valuation of Ecosystem Services and Tradeoffs (InVEST) model is a powerful tool that can be used to reduce subjectivity to quantitatively assess ecosystem services [28]. Morphological spatial pattern analysis (MSPA) is based on graphical principles and combines a raster algorithm to identify ecological sources at the pixel level scale [29,30], emphasizing structural connectivity and thereby increasing the scientific validity of ecological source selection [31]. Based on the goal of comprehensively enhancing ecosystem services and landscape connectivity, the combined use of the above two methods will allow for a more scientific and objective identification of ecological sources, better reflecting biological conservation needs and the suitability of species habitats. The creation of ecological resistance surfaces is another core element of ecological network establishment. The evaluation framework has developed from simply assigning values to land use types to considering human interference activities, and has become multi-faceted and more objective. Ecological corridors, as bridges between ecological patches, are linear or banded landscape elements different from the substrate on both sides and channels for exchanging materials, energy, and information between ecological patches. The minimum cumulative resistance (MCR) model proposed by a Dutch ecologist [32] has been optimized by Yu [33] and has been widely used in the extraction of ecological corridors. The model reflects the ease of “source” dispersal by the magnitude of cumulative resistance, which can effectively reflect the potential possibility and trend of species and energy flow and dispersal in the region. Circuit theory, which applies the random travel of electrons in a circuit to simulate the diffusion of species or ecosystem services within a region [34], can reflect the relative importance of ecosystem services in patches and corridors and identify key nodes in the flow process, and is also widely used in related studies [35,36,37]. Others have used the Linkage Mapper tool integrating the least-cost path (LCP) method and Circuit theory to extract ecological corridors [38,39]. The Linkage Mapper tool first identifies adjacent ecological sources, then constructs a network between ecological sources by adjacency and distance from each other, then calculates the cost-weighted distances and least-cost paths between different source locations, and finally combines the least-cost corridor links that need to be connected into a map [40]. However, any resulting corridors should not only be a conceptual connecting path, but should also have width. Effective corridors are those that can effectively link ecological space of wildlife with land use policies [41]. The corridor width setting level should be combined with the movement characteristics of the research object in order to achieve a balance between the purpose of protecting the ecological environment and avoiding a large conflict with economic development [17,42,43]. Ecological nodes are critical and strategic nodes that occupy important locations on ecological corridors, generally located at the weakest part of the corridor [44], and they play an important role in improving the connectivity of the existing ecological network and promoting healthy ecosystem operation [45,46].

In general, the current studies on ecological network mainly focus on different scales such as provincial areas, urban clusters, cities, and counties [47,48] and typical objects such as mining areas, plateaus, plains, and watersheds [49,50,51], while mountainous areas are less studied. Nearly 65% of China’s land area is mountainous [52]. Mountainous areas have complex topography, which affects the transfer of materials and energy and constitutes a unique ecological environment with important ecological security maintenance functions but a fragile ecological substrate, and is thus a representative area for spatial control studies. The mountainous areas of Southwestern Hubei Province (MASHP) are located in the hinterland of Hubei Province. It is a superimposed zone of Hubei Province and even China’s key ecological function zone, ecological water conservation zone, biological resource enrichment zone, key link zone between poverty alleviation and rural revitalization, and key ecological tourism development zone. The Three Gorges project is located in Sandouping Town, Yichang City, in the MASHP. The MASHP is a typical mountainous area with important ecological location, fragile ecological environment, and intertwined ecological problems and poverty problems, and the contradiction between economic and social development and ecological environmental protection is increasingly prominent [53]. The Nineteenth National Congress of China Report (2017) clearly states that the quality and stability of ecosystems can be effectively improved by building ecological corridors and biodiversity conservation networks. However, with the comprehensive promotion of ecological civilization in China, under the constraint of laws and regulations such as the Yangtze River Protection Law of the People’s Republic of China and the implementation of strategies such as “to step up conservation of the Yangtze River and stop its over development” and “Green Enshi” [54], the governments in the MASHP have made substantial efforts to rectify the ecological problems and promote the implementation of joint prevention and control mechanisms and co-management and co-construction models between various regions, and the ecological environmental protection has achieved phased results. However, the contradiction between ecological environment and economic development is still prominent, and the situation of ecological and environmental security is still serious. Therefore, it is typical and urgent to study the construction and optimization of ecological networks and their protection and restoration in the MASHP.

The MASHP in central China was selected as the study area in this study. The specific research objectives are as follows: (i) based on the integration of ecosystem service hotspots and the MSPA method, the minimum area threshold is set scientifically and combined with landscape connectivity analysis to propose a new comprehensive method for identifying ecological sources; (ii) the ecological corridors are extracted based on the least-cost paths, and the intersection of the maximum paths and the minimum paths are identified as the ecological nodes; (iii) ecological sources, ecological corridors and ecological nodes of different importance levels together constitute the final ecological network; (iv) the internal optimization measures of the ecological network are proposed. The research results can provide the scientific basis for ecosystem restoration, ecological security, territorial spatial planning and high-quality economic development in the MASHP and provide reference for the construction of ecological network and comprehensive ecological restoration and management in other mountainous areas in China.

## 2. Materials and Methods

### 2.1. Study Area

**Figure 1:**
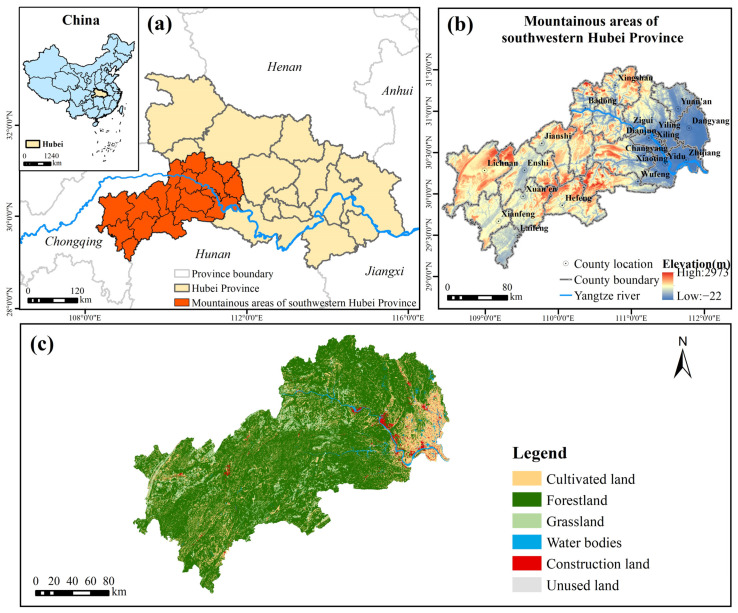
Location of the study area: (**a**) location in Hubei Province, China; (**b**) digital elevation map (DEM); (**c**) land cover.

The MASHP is located in the southwest hinterland of Hubei Province, China, east of the Yunnan–Guizhou Plateau and west of the Jianghan Plain, at 108°22′ E–112°05′ E, 29°08′ N–31°35′ N, including Yichang City (with 5 districts, 3 cities and 5 counties) and Enshi Tujia and Miao Autonomous Prefecture (Enshi Prefecture, with 2 cities and 6 counties) (Figure 1). The MASHP covers a total area of about 45,113 km^2^ and a population of 7,921,200 (in 2020). The mountainous area is undulating and dominated by high and mid-alpine mountains, with an average elevation of over 900 m and a maximum of over 2900 m. The MASHP has a humid subtropical monsoon climate with abundant rainfall. The mountains are crisscrossed by rivers and deep river valleys, with the Yangtze River and Qingjiang as the main water systems. The area is rich in forest resources; vegetation types are coniferous forest, evergreen broad-leaved forest and shrub; rare plant and animal resources are very rich. The MASHP is a very important ecological refuge and gene pool for plants and animals in Hubei Province and even the whole central region of China. In recent years, with the rapid socio-economic development, the expansion of construction, green land crowding, tourism development and large-scale rural deforestation, dominant agricultural development, as well as the construction of dams and other water conservancy facilities in the MASHP, have caused more serious soil erosion, environmental pollution and ecological degradation, and ecological and environmental problems are increasingly prominent.

### 2.2. Data Sources and Processing

The data information used in this study is shown in Table 1, which mainly includes land use data, digital elevation model (DEM), traffic and river data, meteorological data, soil data and normalized difference vegetation index (NDVI), and the above data were processed to obtain the indirect data needed to be used in this study. ArcGIS 10.2 software (ESRI,2013,10.2) (Environmental Systems Research Institute, Redlands, CA, USA) was used to project, pre-process, map and analyze the data in this study. All data were unified in the Albers coordinate system, and the grid of the raster data was unified at 30 m × 30 m. Among them, the land use data were classified into 6 primary types and 25 secondary types according to the land use/land cover changes classification standard, which are defined in Table S1 in Supplementary Materials.

### 2.3. Method

**Figure 2:**
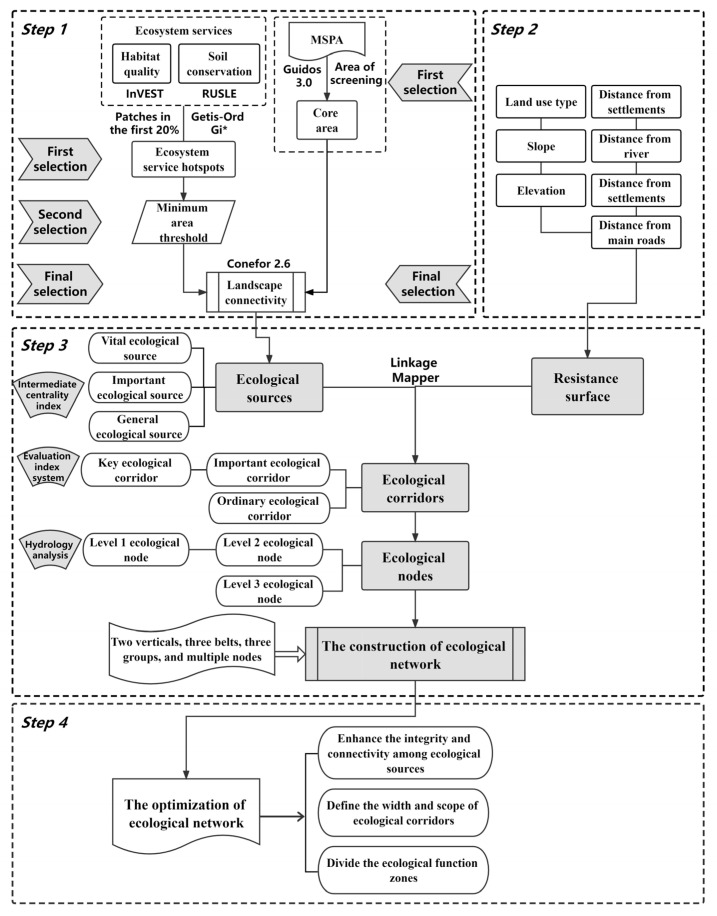
Framework of this study.

Certain flow paths (corridors), critical junctions (nodes) and localities (sources) or their spatial combinations together constitute the ecological network structure, which is divided into four steps: (1) identifying the ecological sources; (2) constructing the resistance surface; (3) extracting the ecological corridors and ecological nodes; (4) establishing the ecological network and optimization. The research framework is shown in Figure 2.

#### 2.3.1. Identification of Ecological Sources

In this study, the final ecological sources were identified by integrating the assessment of two ecosystem service hotspots, namely habitat quality and soil conservation, with the MSPA method, setting the minimum area threshold scientifically and analyzing the landscape connectivity. The specific steps are:

(1) Assessment of ecosystem services

For the development positioning of the MASHP as an important ecological barrier in the middle and upper reaches of Yangtze River, as well as its ecological background situation and related regional management objectives (mainly for soil erosion), two ecosystem services, habitat quality and soil conservation, were selected for quantitative evaluation, and the top 20% patches of each ecosystem service were selected and taken as the first part of alternative ecological sources after merging.

Habitat quality indicates the potential of the ecosystem to provide an environment for species to survive and thrive, and it is positively correlated with biodiversity, thus characterizing the richness of biodiversity to some extent. In the Habitat Quality module of the InVEST model (https://naturalcapitalproject.stanford.edu/software/invest, (accessed on 31 March 2022)), the habitat quality in the MASHP is quantitatively assessed in terms of external threat factors and habitat sensitivity [55,56,57]. This study was based on reference studies of similar areas [58,59], determined the threat factors of habitats, the impact range and weight of each threat factor, the suitability of different types of habitats and their relative sensitivity to different threat factors (Tables S2 and S3). The closer the habitat quality is to 1, the higher the level of habitat quality is indicated, and the calculation formula is as follows:

(1) $$Q_{xj}=H_{j}\left[-\left(\frac{D_{xj}^{z}}{D_{xj}^{z}+K^{z}}\right)\right]$$

where *Q_xj_* is habitat quality of raster *x* in land use type *j*, *H_j_* is the habitat suitability of land use type *j*, *D_xj_* is the habitat degradation of raster *x* in land use type *j*, *K* is the half-saturation constant, which is generally taken as half of the maximum value of *D_xj_*, and *Z* is the normalization constant, which is the default parameter of the system and usually takes the value of 2.5.

The modified universal soil loss equation (RUSLE) [60] was used to estimate the potential soil loss without considering vegetation cover factors and soil and water conservation measures and the actual soil loss considering the factors mentioned above. The difference between the two was taken as the soil conservation amount of the ecosystem [61,62]. The calculation formula is as follows:

(2) SC=R×K×L×S×(1−C×P)

where *SC* is the amount of soil conservation (t·hm^−2^·a^−1^), *R* is the rainfall erosion force factor (MJ·mm·hm^−2^·h^−1^·a^−1^), *K* is the soil erodibility factor (t·h·MJ^−1^·mm^−1^), *L* and *S* are the topography factors (*L* is the factor of slope length, *S* is the slope factor), *C* is the surface vegetation cover factor, and *P* is the soil conservation measure factor.

(2) Application of hotspots analysis

The spatial distribution of cold and hot areas for ecosystem services reflects the strength of ecosystem services. The hotspot analysis method is based on the Getis-Ord Gi* statistical method [63], which analyzes the spatial clustering of high or low values of ecosystem services. Patches that are fragmented and do not have concentrated contiguity are continuously eliminated as the distance threshold increases, and close and connected patches are clustered to form larger patches. Based on the evaluation results of habitat quality and soil conservation, the top 20% patches of each ecosystem service were selected to select ecological service hot areas according to extreme confidence hot areas, and the first round of selection of the first part of alternative ecological sources was completed.

(3) MSPA

This study identified and extracted the core area in the MASHP based on the MSPA method as the second part of alternative ecological sources [30]. Considering the geographical conditions of the MASHP, forestland was set as foreground and other land use types were set as background. There is an obvious scale effect in the MSPA method, and the identification results are not consistent with different threshold values [64]. For example, if the threshold is too large, elements with smaller areas will disappear or be classified under other elements, and small core areas will be classified as isolated islands, etc. Therefore, the threshold value was set to 100 m × 100 m by considering the effects of the area, data, and scale effects of the study area. Finally, the data were binarized, rasterized, and analyzed in Guidos Toolbox 3.0 analysis software (https://forest.jrc.ec.europa.eu/en/activities/lpa/gtb/, (accessed on 31 March 2022)). The data were analyzed using the eight-neighborhood analysis method to obtain seven types of non-overlapping landscapes, namely, Core, Islet, Perforation, Edge, Loop, Bridge, Branch and Background (see Guidos Toolbox user guider for detailed meanings of landscape types and color symbols) [65]. Finally, core areas that were important for maintaining connectivity were extracted as landscape elements for subsequent analysis. In order to avoid fragmented patches from degrading the main function of the core area and generating redundant ecological corridors, the patches with an area of not less than 10 km^2^ were selected for the subsequent patch connectivity calculation with reference to the studies on the threshold setting of relevant core area by MSPA [28,66]. The first round of selection of the second part of alternative ecological sources was completed at this time.

(4) Evaluation of landscape connectivity

Landscape connectivity reflects the degree to which the landscape facilitates or impedes ecological flows, has a positive impact on species richness and is key to maintaining ecosystem stability and integrity [67,68]. Based on the Conefor Sensinode 2.6 software (Jenness Enterprises, Flagstaff, AZ, USA) (http://www.conefor.org/coneforsensinode.html (accessed on 31 March 2022)), starting from the probability of connectivity (*PC*) and the integral index of connectivity (*IIC*) reflecting the connectivity of each patch to the landscape, the delta values for the *PC* index (*dPC*) and the Delta Values for the *IIC* index (*dIIC*) of alternative ecological source patches were calculated. The average value (*dI*) of the two was taken to obtain a more accurate degree of ecological patch importance [69]. A higher index means a higher degree of patch connection. The node files and distance files required for connectivity analysis in Conefor Sensinode 2.6 were generated by the plug-in for Conefor in ArcGIS 10.2 software and were calculated as follows:

(3) $$PC=\left(\sum_{i=1}^{n}\sum_{j=1}^{n}a_{i}\times a_{j}\times P_{ij}^{*}\right)/A_{L}^{2}$$

(4) $$IIC=\sum_{i=1}^{n}\sum_{j=1}^{n}\left[\left(a_{i}a_{j}/\left(1+nl_{ij}\right)\right)\right]/A_{L}^{2}$$

(5) $$dPC=\left(PC-PC_{remove}\right)/PC\times 100\%$$

(6) $$dIIC=\left(IIC-IIC_{remove}\right)/IIC\times 100\%$$

(7) dI=0.5×dPC+0.5×dIIC

where *n* is the total number of patches in the study area, *a_i_* and *a_j_* are the areas of patch *i* and patch *j*, respectively, *P_ij_* is the maximum probability of species dispersal between patches *I* and *j*, *A_L_* is the total landscape area in the study area, *l_ij_* is the shortest path from patch *I* to patch *j*, *PC_remove_* is the *PC* after removing an element in the landscape, and *IIC_remove_* is the *IIC* after removing an element in the landscape.

(5) Final confirmation and optimization of ecological sources

The first part of alternative ecological sources should be of a specific size to ensure the stability of ecosystem service provision and the positive aggregation effect [70]. This study quantitatively determined the minimum area threshold of ecological sources by exploring the variation of the number of patches and the total area of patches with the selected minimum area threshold, and completed the second round of selection of the first part of alternative ecological sources. The two previously obtained parts of alternative ecological sources were combined after removing the small overlapping parts, and the *dI* values of the patches were calculated. Finally, the patches with *dI* greater than 2 were identified as the final ecological sources, and the final round of selection of the first and second parts of alternative ecological sources was completed at this time.

The intermediary centrality index (0< *Q_i_* ≤ 1, the closer *Q_i_* is to 1, the greater the importance) was calculated to define the role of ecological sources, and the results were classified qualitatively: *Q_i_* ≥ 0.5 is classified as vital ecological source; 0.2 < *Q_i_* < 0.5 is classified as important ecological source; 0 ≤ *Q_i_* ≤ 0.2 is classified as general ecological source; the formula is as follows:

(8) $$Q_{i}=\frac{dPC_{connector}}{dPC}$$

In the equation, *dPC_connector_* quantifies the importance of patch *i* in maintaining the overall effective connection in the ecological network, and *dPC* quantifies the maximum flux through patch *i* in the complete landscape diffusion process.

Considering the increased urban expansion and demand for ecosystem services in the future, the current ecological resources may not be sufficient to meet the needs of future development, so the ecological sources need to have a wider radiative power for maintaining the ecological processes and natural succession within the sources and reducing the impact of anthropogenic disturbances in the external landscape [71]. Since the MASHP is rich in forestland resources, forestland with the strongest ecosystem services was selected as the main factor influencing the spread of ecological sources [72]. In this study, multiple ring buffer zones (100, 200, 300, 400, 500, 600, 700, 800, 900, and 1000 m) were established for ecological sources with poor landscape connectivity (2 ≤ *dI* < 3), and the change in forestland growth rate in each buffer zone with the increase in buffer distance was analyzed. Finally, the optimal diffusion distance of these sources was obtained by integrating the distribution of other land use types.

#### 2.3.2. Determination of Resistance Surface

When determining ecological resistance, referring to the experience of previous studies [46,58,73], a five-level system was used to define the resistance magnitude of a single factor, with higher scores implying greater species dispersal resistance. The AHP method was used to determine the resistance factor weights. The weights and coefficients of the resistance factors are shown in Table 2.

#### 2.3.3. Extraction of Ecological Corridors and Ecological Nodes

In this study, the Linkage Mapper tool (https://linkagemapper.org/, (accessed on 5 April 2022).) in ArcGIS 10.2 software was used to extract ecological corridors. First, the cost-weighted distances (CWDs) from each pixel on the ecological resistance surface to the ecological sources were calculated; second, the least-cost paths (LCPs) between all ecological sources were determined; finally, the cut-off distances were set to generate ecological corridors, so that each source was connected and formed a network loop. In view of the identified corridors, an evaluation index system was established to evaluate the importance of corridors by comprehensively considering their functional importance and their own conditions [74]. The functional importance of the corridors included the area of the source patch connected by the corridor and the landscape connectivity of the main source connected by the corridor; the corridor conditions mainly considered the corridor length and corridor quality. Using the AHP method to determine the weights of indicators at all levels, all indicators were divided into three levels using the natural breaks method in ArcGIS 10.2, and the importance was assigned to 5, 3 and 1 from the largest to the smallest, respectively (Table 3). Finally, the ecological corridors were classified into key ecological corridor, important ecological corridor and ordinary ecological corridor according to the calculation results, from the largest to the smallest.

Strengthening the ecological environment of ecological nodes is conducive to reducing the consumption cost of ecological corridors and enhancing the ecological service function of the regional ecological network. In this study, the ecological corridors were the minimum cost distance channels between adjacent sources, namely, the minimum paths. The “ridge lines” of the minimum cumulative resistance surface were obtained in ArcGIS 10.2 using hydrology analysis module, namely, the maximum paths. In the hydrological analysis module of the spatial analysis tool of ArcGIS 10.2 software, the maximum threshold value that blocked ecological flow and species dispersal was extracted based on the minimum cumulative resistance surface, and then vectorized and smoothed the vectorized lines to obtain the “ridge lines” of the resistance surface [75,76]. This study identified the intersection of the maximum and minimum paths as ecological nodes. According to their intersection, the ecological nodes were divided into three levels according to their intersection with corridors of different importance (Table 4).

## 3. Results

### 3.1. Spatial Patterns of Ecological Sources

#### 3.1.1. Assessment of Ecosystem Services and Analysis of Hotspots

**Figure 3:**
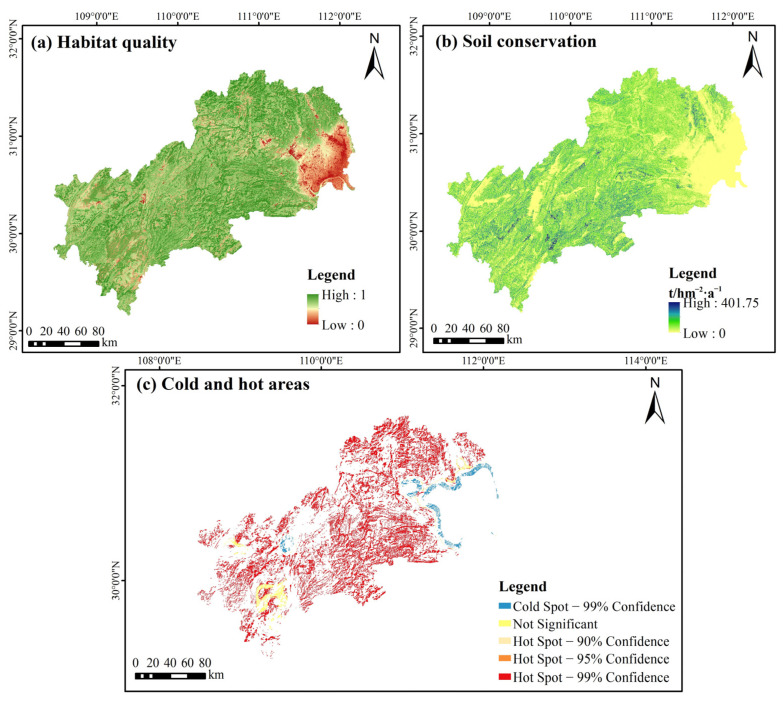
Spatial pattern of ecosystem services and identification of cold and hot areas of ecosystem.

The spatial patterns of the two ecosystem services in the MASHP were obtained by quantitatively evaluating habitat quality and soil conservation ecosystem services. As can be seen from Figure 3, the average habitat quality index in the MASHP is 0.75, which is at a moderate to a high level. Because of the serious fragmentation of the study area, the areas with various levels of habitat quality show a staggered distribution throughout the study area. The areas with higher habitat quality are spread, mostly covered with forest vegetation, and have a good natural environment. They are less disturbed by human activities such as urban land expansion and construction, so the habitat quality indices are high. In terms of soil conservation, the values in the southwest are generally higher than those in the northeast, and the low value areas are mainly located in low elevation areas with less topographic relief, mainly including Zhijiang City and Dangyang City, where the land use types are mainly cultivated land and construction land, with sparse vegetation and high intensity of human activities. In general, the natural background state of the MASHP is good, and the high-value areas of the two ecosystem services mostly overlap with forestland and grassland.

**Figure 4:**
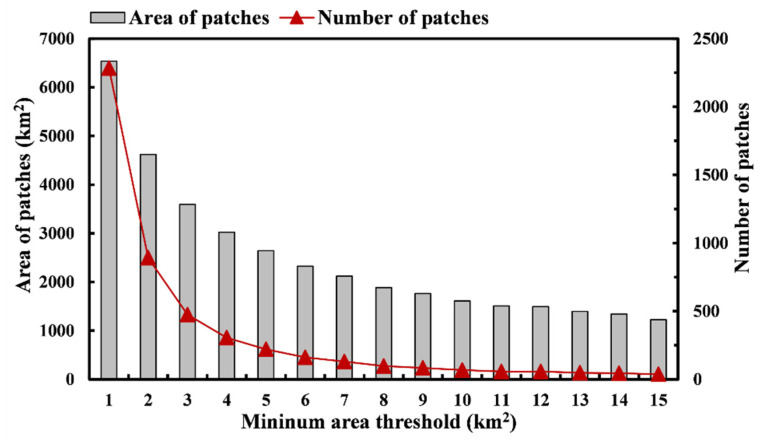
Effect of the minimum area threshold on the number of patches and area of patches from 1 to 15 km^2^.

The spatial distribution of cold and hot areas of ecosystem services reflected the strength of ecosystem services. The evaluation results of habitat quality and soil conservation are integrated, the top 20% ecological patches of each ecosystem service are selected, and the hot areas of ecosystem services in the MASHP are obtained based on ArcGIS 10.2 software after taking the merged set and using the hotspots analysis tool in the spatial statistics module, with extreme confidence hot areas as the selection criteria (Figure 3c). The hot areas are mainly located in the northern, central, and southwestern regions, while the cold areas are concentrated in the eastern region. For the ecological sources selected by hotspots analysis, there are problems with the excessive number of ecological source patches and high fragmentation, so it is necessary to set the minimum area threshold manually to correct them further to avoid the fragmented patches from reducing the ecosystem service function of the sources [48,71,77]. As the minimum patch area threshold of ecological source patches increases from 1 to 15 km^2^ in steps of 1 km^2^, the number and area of patches first show a sharp decline, and then there is a steady and apparent decrease at the threshold of 10 km^2^. At the inflection point, the downward trend of the curve is flat, and there is no longer a vast fluctuation (Figure 4). The fluctuation of the above data indicates that it is better to use the threshold value of 10 km^2^ to eliminate small and finely fragmented patches. Although there are a large number of removed patches, they are small in individual area, finely fragmented and scattered, and have little impact on the regional ecological environment. Therefore, patches with areas larger than 10 km^2^ were selected. 68 patches become alternative ecological sources, and most of these alternative ecological sources are concentrated in the northern and central parts of the study area, and scattered in the southwestern part (Figure S1).

#### 3.1.2. Identification of Core Ecological Patches by MSPA

**Figure 5:**
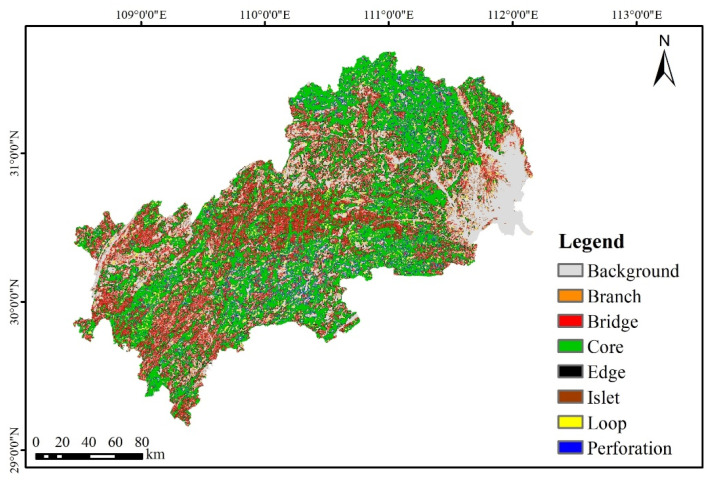
MSPA-based landscape feature type map.

The MSPA patterns distribution of the study area is shown in Figure 5. Among the seven landscape types in the foreground, the core area has the largest area of 16,750.84 km^2^, accounting for 49.98% of the foreground area. Meanwhile, due to the serious fragmentation of patches in the core area, the selection of too many ecological sources will lead to excessive overlapping and redundancy of subsequent ecological corridors, which will increase management costs. Combining with the actual situation of the study area, 103 patches with an area of more than 10 km^2^ in the core area were selected as the alternative ecological sources (Figure S2).

**Figure 6:**
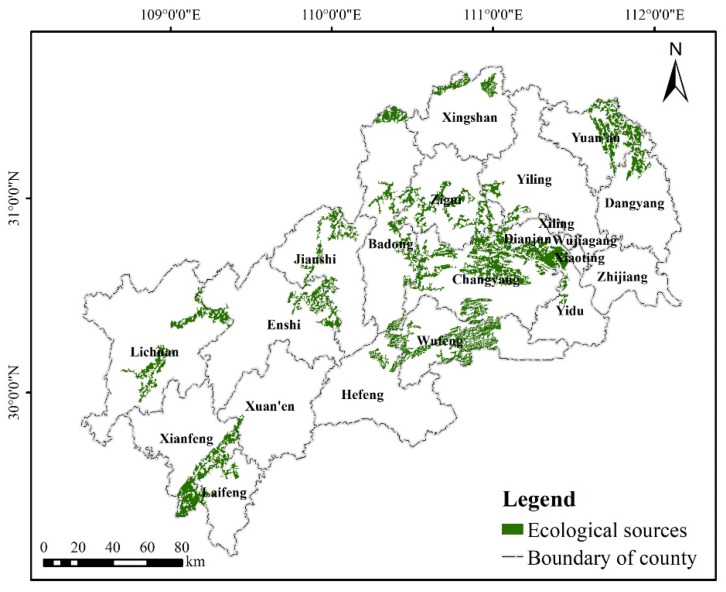
Ecological sources in the study area.

In order to avoid the phenomenon of patch overlap, the two previously obtained alternative ecological source patches were combined after removing the small overlapping parts. Finally, 171 alternative ecological source patches were obtained. Based on the results of previous studies and Equations (3)–(7), the landscape connectivity of 171 patches was assessed using Conefor Sensinode 2.6 software, setting the patch connectivity distance threshold to 1500 m and the probability of connectivity to 0.5, with 68 patches based on ecosystem service evaluation, hotspots analysis and area screening and 103 patches based on MSPA and area screening. The evaluation results show that the patches with *dI* values less than 2 are isolated and small compared to other patches, and the landscape connectivity is poor, so they should not be identified as ecological sources. Therefore, 49 patches with *dI* values greater than 2 were identified as the final ecological sources. The total area of ecological sources is 3837.92 km^2^, accounting for 8.51% of the total area of the MASHP, and the land use types are mainly forestland. Overall, most of the ecological sources are concentrated in the central region, the county boundary areas in the north and south, and scattered in the southwest, with the number of sources gradually decreasing from the center to the two ends, and the landscape connectivity in the southwest is poor (see Figure 6 and Figure S3). Some of the larger ecological sources (e.g., number 22, 38) are close to the study area boundary, while other relatively small ecological sources (e.g., number 9, 42) are mainly in the central part. From the spatial distribution of ecological sources by county, Changyang Tujia Autonomous County has the largest proportion of ecological sources in the total county area (19.95%), followed by Wufeng Tujia Autonomous County (10.43%). In contrast, Xiling District, Wujiagang District, Xiaoting District and Zhijiang City have no ecological source distribution. As shown by the overlay analysis with various national nature reserves of the MASHP (http://www.papc.cn/html/folder/1-1.htm (accessed on 1 May 2022)), most of the ecological sources obtained involve the core areas of existing nature reserves and forest parks, such as Xingdoushan National Nature Reserve (in Lichuan), Mulinzi National Nature Reserve (in Hefeng), Houhe National Nature Reserve and Chaibuxi National Forest Park (in Wufeng), indicating that the selected sources are scientific.

Based on Equation (8), *Q_i_* was calculated to evaluate the importance of each ecological source patches, so as to determine the connotation of ecological source patches in regional potential ecological network. Finally, there are 14 vital ecological sources, 12 important ecological sources and 23 general ecological sources.

### 3.2. Analysis of Comprehensive Resistance Surface

**Figure 7:**
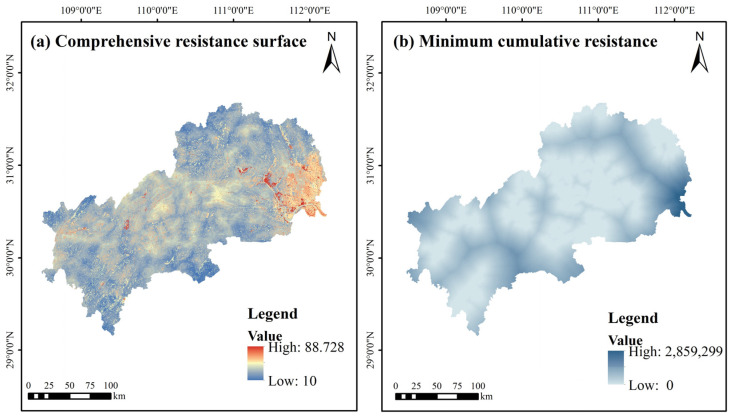
Spatial distribution of comprehensive resistance surface and minimum cumulative resistance.

According to the resistance factors and weights in Table 2, ArcGIS 10.2 was used to establish the comprehensive resistance surface of ecological factors. The minimum cumulative resistance surface was generated based on ecological sources and comprehensive resistance surface to lay the foundation for the subsequent delineation of function zones. The average resistance value is 34.52, and the comprehensive ecological resistance surface shows an overall staggered distribution of high and low resistance values, with a certain aggregation in some areas (Figure 7a). The high-value ecological resistance areas show the characteristics concentrated in the northeast and scattered in the rest of the area, basically located in the areas with the high level of urban development, high traffic density and frequent human activities. Although cropland has vegetation with good growth, its resistance value rises due to the influence of human agricultural activities and artificial control around it. Topographic obstruction factors also influence the high value of resistance areas distributed in Enshi Prefecture, and the high terrain makes the spread of ecological species narrow. The low-value ecological resistance areas are located in the north, south and southwest, where the surface vegetation coverage is high, there are rivers and lakes, and the anthropogenic interference is less than that in the urban concentration. From Figure 7b, it can be seen that some areas in the eastern, southern and western parts of the study area can have a more significant obstructive effect on the migration dispersal and material flow of species.

### 3.3. Analysis of Ecological Corridors and Nodes

**Figure 8:**
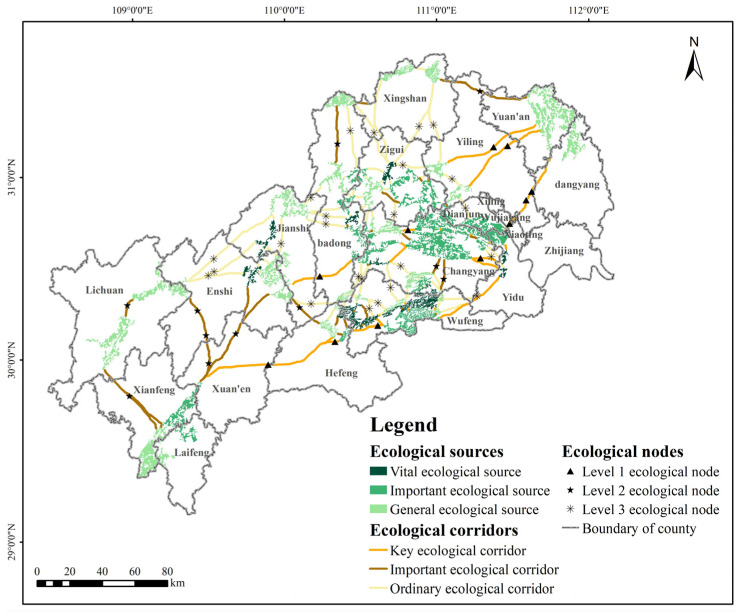
Ecological network in the study area.

The Linkage Mapper tool was used in ArcGIS 10.2 to extract the LCPs to complete the ecological corridor identification based on the ecological sources and comprehensive resistance surface. In this study, 125 ecological corridors were identified, with a total length of about 2014.61 km. The ecological corridors are connected along the northeast–southwest direction to form a network and run through the whole area, with a dense distribution in the central part, indicating that the central source areas are better connected. Most of the corridors spreading from the east and west ends to the middle are long-distance corridors. Affected by the barrier of urban built-up areas, there is a potential connection trend between some sources separated by distance, but no connected corridor has been formed (Figure 8). After analyzing the statistics, it is found that the corridor length distribution in three regions, Changyang Tujia Autonomous County, Xuan’en County and Enshi City, shows obvious advantages, with a total of 640.11 km, accounting for about 31.77% of the total length of ecological corridors. The corridors show an extensive spanning range in these areas. There are no ecological corridors distributed in Xiaoting District, Zhijiang City and Laifeng County, mainly for three reasons: firstly, most of the construction land in the above counties is centrally distributed, with high ecological resistance values and large barriers to communication between ecological sources; secondly, regional construction land is distributed sporadically among ecological patches, and ecological patch fracture cannot form a good ecological concentration surface; lastly, there are regions with large areas of contiguous cultivated land, without ecological source land coverage. According to Table 3, the importance of each ecological corridor was calculated. Finally, there are 26 key ecological corridors, 29 important ecological corridors, and 70 ordinary ecological corridors with total lengths of 543.80 km, 536.56 km, and 934 km, specifically. Key and important ecological corridors are important paths for the diffusion of ecosystem services from the sources to the outside, ensuring the fundamental ecological processes. Additionally, some corridors are also the only corridors for circulation between some ecological sources, with a wide range of radiation. However, because of the long length of some corridors, they are susceptible to anthropogenic impacts such as urban expansion, excessive agricultural settlement and vegetation destruction, so there is an urgent need to optimize the corridors in terms of adjusting the spatial layout of functions and increasing the density of regional vegetation cover. Ordinary ecological corridors are supplementary to other level corridors, forming a north–south coherent ecological network.

The number, quality, and distribution of ecological nodes affect species’ migration timing and success probability. A total of 46 potential ecological nodes were identified in the study area, including 11 level 1 ecological nodes, 11 level 2 ecological nodes, and 24 level 3 ecological nodes (Figure 8). These nodes mainly play the role of connections and hubs, located at the weak point of ecological material and capacity exchange, which are crucial for the mobility efficiency of the ecological corridor and greatly influenced by human activities [17]. The ecological nodes identified are relatively evenly distributed spatially, in total, on 42 ecological corridors. Still, no ecological nodes are distributed in Zhijiang City, Dangyang City, and Yuan’an County in the east and Laifeng County at the southern end. The landscape type of most ecological nodes is forestland, but any two points are far apart and poorly connected between nodes, which also indicates the need to increase ecological nodes in areas with dense source distribution to promote connectivity and strengthen the construction and protection of ecological nodes. Level 1 ecological nodes are scattered and relatively few in number, and there is no distribution in the north. They play an important role in connecting ecological sources and assume the responsibility for smooth operation of ecological flow and maintenance of ecological stability. Therefore, the preservation and optimization of the original green belt and water patch with high ecological function in the area where the nodes are located should be strengthened. Level 2 ecological nodes are more distributed in the southwest, and some of them are also in the transition zone between urban construction land and cultivated land. Landscape transformation of such nodes has high cost and difficulty, so local governments should focus on improving the ecological efficiency of green land and enhancing the ability to prevent pollution. Level 3 ecological nodes are the most numerous, mostly covering the central and northern parts of the study area. Three of them are cultivated land, vulnerable to human development activities. Moreover, the spread of agricultural non-point source pollution can greatly interfere with the circulation of ecological flow. In the future, the regional government can consider constructing “stepping stones” at ecological nodes by building small ecological parks or planting forestland to improve ecological network connectivity and provide landing points for biological migration.

### 3.4. Construction of Ecological Network

Ecological corridors are interwoven with ecological nodes together with ecological sources to form ecological network. The ecological network in the MASHP includes 49 ecological sources, 125 ecological corridors, and 46 ecological nodes, all of which have different levels of importance. Ultimately, these elements form a sustainable, composite, multi-level and interconnected ecological network (Figure 8). In this ecological network, the coverage of ecological sources is ideal, ecological corridors can effectively link the sources, and ecological nodes play an important role in promoting the flow of energy and material in the region.

**Figure 9:**
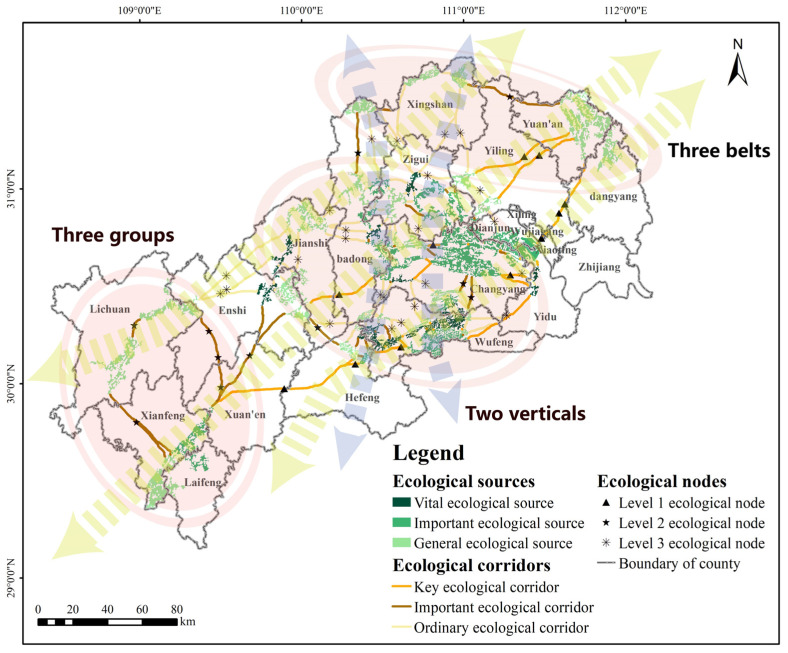
Ecological framework in the study area.

Combining the spatial distribution characteristics of elements, the ecological spatial framework of the MASHP can be summarized as “two verticals, three belts, three groups, and multiple nodes” (Figure 9). The “two verticals, three belts” represent the general direction of ecological corridors of a certain scale connecting all ecological sources and nodes, mainly the vertical ecological corridors starting from Badong County and Xingshan County and the longer horizontal ecological corridors in the northeast–southwest direction. The ecological corridors expressed as vertical axes maintain the connectivity between the north and the central and southern parts. In contrast, the ecological corridors expressed as horizontal axes link the ecological sources at the northeast and southwest ends with the central sources, ensuring the integrity and continuity of ecological processes from north to south and from east to west, which are the best paths for species migration and ecological information flow. The “three groups” represent the northeastern, central, and southwestern ecological groups, respectively. The northeastern ecological source group mainly includes ecological sources distributed in the northern end of Badong County, Xingshan County and Yuan’an County, mainly at the edge of the county boundary. This group is responsible for important ecological functions such as soil conservation and biodiversity maintenance northeast of MASHP. The central ecological group includes ecological sources mainly in Changyang Tujia Autonomous County and Wufeng Tujia Autonomous County, which is the core group. The ecological sources in this group are concentrated, crossed by several ecological corridors, rich in species and with good natural substrates. The southwest mainly includes ecological sources in Lichuan City, Xianfeng County and Laifeng County, which is the main ecological source group in Enshi Prefecture. The source patches are large in area but small in number, which guarantee the ecosystem security and stability in the southwestern part of MASHP. The “multiple nodes” represent the ecological nodes of different levels in the network, which are located at the weakest part of the corridor function. The connectivity degree and cross-structure features of the ecological network in the MASHP have noticeable spatial differences, so further details can be improved and optimized from the perspective of improving the stability and liquidity of the overall ecosystem.

## 4. Discussion

### 4.1. Optimization of Ecological Network

The current problems of ecological network in the MASHP created in this study are reflected in three aspects. Firstly, the fragmentation of the landscape in the study area is serious, causing fragmentation of ecological resources, resulting in the uneven spatial distribution of ecological sources and insufficient diversity of ecological elements, for example, some small patches in the central ecological source group are isolated and not well connected. Therefore, according to the spatial structure and distribution characteristics of the existing ecological sources, the related management departments should focus on maintaining and effectively improving the ecological quality and ecological benefits of the ecological sources, enhancing the connectivity between the sources, and dividing ecological protection buffer zones to avoid damage to the ecological sources. Secondly, the existing ecological corridors need to be further widened, and only corridors with a certain width can assume the function of ecological element connectivity and communication. The width of ecological corridors in ecological network optimization can enhance the ecological effect of corridors and the connectivity between small ecological corridors. Therefore, a certain width of ecological corridors should be given to activate the ecological service function of the corridor [68]. Finally, the existing ecological network elements are in conflict with other production and living service elements, and the planned new infrastructure is also prone to damage the ecological network; for example, the completion of new national highways will affect several ecological corridors. Therefore, different ecological functional zones can be divided to set up targeted management and protection measures to effectively coordinate the value balance of agricultural production, development and construction and ecological services.

To further enhance the integrity and connectivity among ecological sources, buffer zones were set for ecological sources with poor landscape connectivity (2 ≤ *dI* < 3), and the growth rate of forestland within each buffer would be observed to change as the buffer distance becomes larger, and finally, the optimal dispersal distance of these sources were obtained after integrating the distribution of other land use types (Table S4 and Figure S4). The analysis of the evolution of the buffer zones of ecological sources in the MASHP shows the following: when the buffer zones of some sources reach a certain distance, they overlap with the surrounding construction land, so this phenomenon should be avoided as much as possible; when the buffer zones of some sources are too large, the buffer zones of ecological source patches overlap each other and start to crowd the space of other lands, which is not consistent with the reality of intensive land use; when the buffer zones of some sources are increased to the corresponding distance, the connectivity with the surrounding sources enhance, but the optimal diffusion distance need to be determined after comprehensive consideration. In summary, the buffer zones are set up for ecological source patches with poor landscape connectivity. As the ecological sources spread outward, some ecological sources will form effective connections, thus enhancing the connectivity between ecological sources and the stability within the patches [71,72]. For instance, patches numbered 14 and 12 and patches numbered 9 and 16 in the central eco-logical source group have connected after installing buffer zones to realize the expansion of outward radiation (Figure S5).

**Figure 10:**
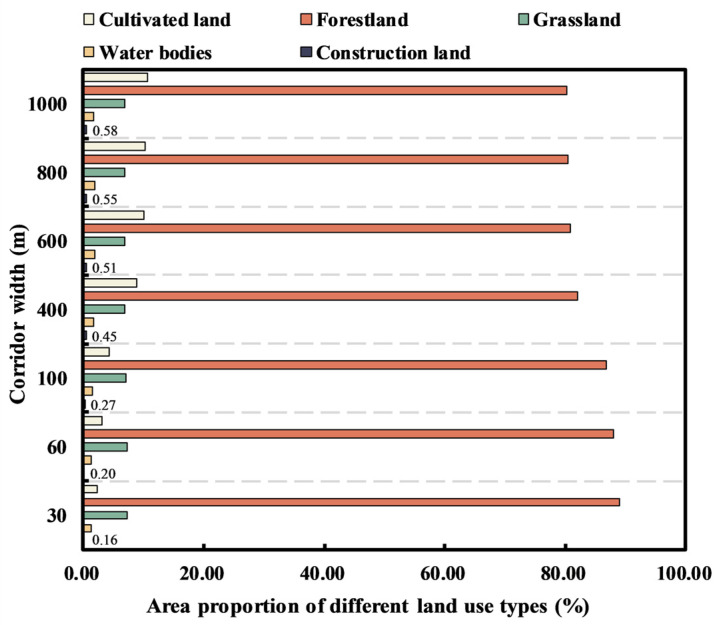
Area proportion of different land use types in corridors with different widths.

The connectivity of ecological network is ensured by dredging ecological corridors. The animal species in the MASHP are mainly medium-sized mammals. According to Zhu et al. [78], buffer zones of 30, 60, 100, 400, 600, 800, and 1000 m were set for existing ecological corridors, and the area of each land use type in different corridor widths was statistically analyzed. As shown in Table 5 and Figure 10, it can be seen that the distribution area of each land use type in different corridor widths varies greatly. Forestland is the primary type in the corridor. As the corridor width increased from 30 m to 1000 m, the area of all land use types shows a continuously increasing trend. Although the proportion of forestland area to the total area of corridor area decreases continuously, the proportion is always more than 80%. The proportion of cultivated land area increases less, and after 400 m corridor width, the proportion of cultivated land area to the total area of corridor area is second only to forestland area. The proportion of grassland area increases, then decreases and then increases again but remains stable at 7% or less, with the inflection points occurring at 60 m and 600 m corridor widths, respectively. The proportion of water bodies area increases and then decreases, with the inflection point and the highest point occurring at the 600 m corridor width. The proportion of construction land area keeps rising, but it is always below 1%, and the increase rate increases significantly to 400 m. It is worth noting that the large construction land area is not conducive to the migration and diffusion of organisms between ecological sources. It also increases the difficulty of constructing ecological corridors. Taking all factors into consideration, the corridor width of 100–400 m has the least encroachment on cultivated land, weak impact on construction land, and the area share of three major ecological land use types, namely, forestland, grassland and water bodies, is at a high level, which is also the width required for migration and conservation of small- and medium-sized mammals [79]. Therefore, the optimal width range of the MASHP corridor is determined to be 100–400 m. In the future construction of the corridors, based on the natural advantages of forestland and grassland in the study area, the stability of the corridors should be enhanced by forming a vegetation community with a composite structure of trees, shrubs, and grasses according to the actual situation and by strengthening the configuration of ecologically strong and stable vegetation. In particular, when building long and important ecological corridors, the distance between core patches can be shortened by adding “stepping stones” and buffer zones to prevent the rupture of corridors. For corridors that may cross water bodies (for example, some vertical ecological corridors starting from the northern part of the study area will span the Yangtze River and its tributaries), attention should be paid to the construction of coastal vegetation buffer zones of water bodies where the corridors are located. A reasonable vegetation buffer zone can effectively filter surface pollutants to improve water quality and create conditions for the migration of inland organisms in the corridors. For corridors with cultivated land distributed inside, the focus should be on protecting semi-natural habitats such as pond wetlands, ecological ditches, farmland ridges and scrubs, and moderate conversion of farmland back to forests to maintain the ecological stability of the small regional environment. For corridors with a large distribution of internal construction land, a buffer zone of a certain width can be set outside the corridor to minimize the interference of large human construction activities in the areas around the corridor. A portion of ordinary ecological corridors exist between compact ecological sources with short lengths, and such corridors can facilitate further species circulation by enhancing the closeness between ecological sources [74]. Of course, the ecological network is a resilient ecological conservation space in which the width of the corridors can be appropriately contracted and expanded according to the practical problems faced during construction [17].

**Figure 11:**
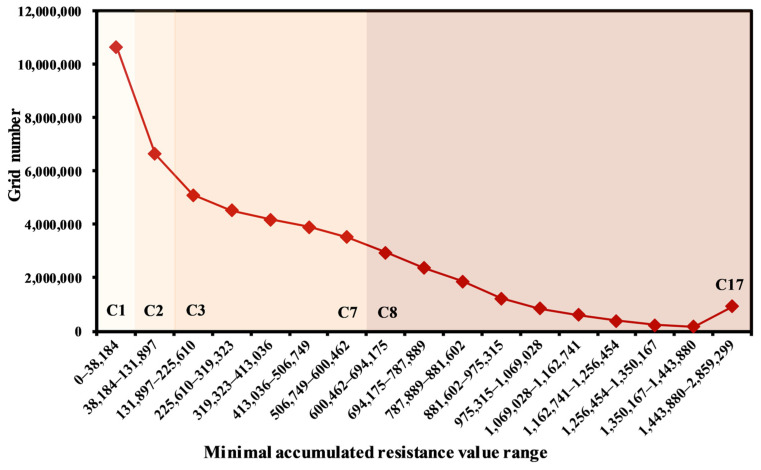
Relationship of grid number and minimal accumulated resistance.

**Figure 12:**
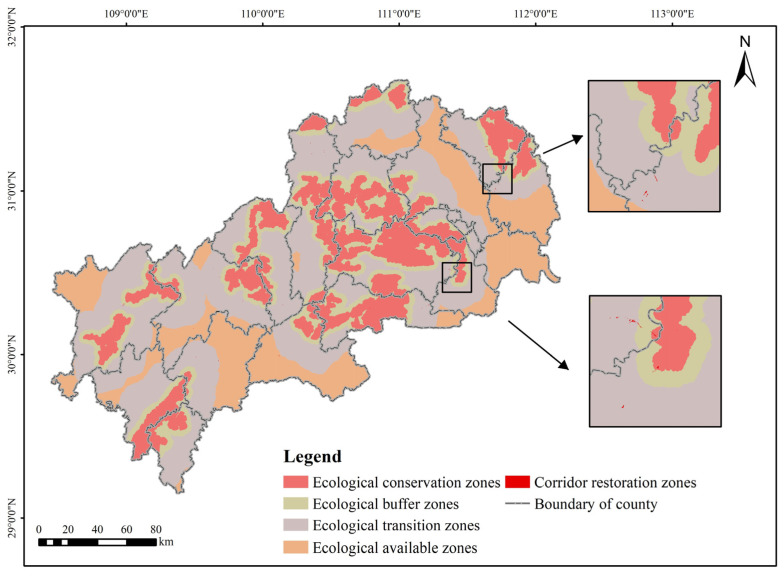
Ecological function zoning based on ecological network.

By dividing the ecological function zones, the precision and efficiency of ecological security construction can be ensured. The minimum cumulative resistance surface constructed based on ecological sources is the basis of ecological function zoning [76]. The standard deviation classification method was used to classify the resistance values. The mutations in the number of grids corresponding to different cumulative resistance numbers were selected as the basis for determining the resistance thresholds. When species pass through these mutation points, the minimum cumulative resistance value will increase sharply, indicating that when the ecological land area increases to a certain extent, the conservation significance of increasing the area again will decrease suddenly. According to the standard deviation of the minimum cumulative resistance, the resistance values of ecological land protection were initially divided into 17 categories, named C1–C17, and each category was separated by one-quarter variance. The number of grids in each category and the correspondence of the image element value (minimum cumulative resistance value) were counted (Table S5). As shown in Figure 11, from C1–C2, there is a significant decrease in the number of grids, and the difference in the number of grids accounts for 8.02% of the total number of grids in the MASHP; from C3–C7, the number of grids again undergoes a relatively large abrupt change, and the change is second only to that from C1 to C2, accounting for 3.18% of the total number of grids; starting from C8 onward, the number of grids tends to stabilize, remaining at a level below 6%. Therefore, according to the above threshold determination method, the minimum cumulative resistance values of 38,183, 131,896 and 600,462 were selected as the critical threshold functional zoning in the MASHP. At the same time, the spatial distribution patterns of ecological sources, ecological corridors and ecological nodes in the study area were comprehensively considered. The corresponding division results were ecological conservation zones (C1, ECZs), ecological buffer zones (C2, EBZs), ecological transition zones (C3–C7, ETZs), and ecological available zones (C8–C17, EAZs), with the objectives of protection and conservation, stability maintenance, conflict mitigation and production development, respectively. In addition, ecological function zoning also needed to consider the spatial distribution pattern of ecological corridors. The primary function of ecological corridors was to provide species migration and dispersal channels. Therefore, based on the composition of land use types in ecological corridors, the corridor restoration zones (CRZs) with the greatest resistance to species migration and dispersal in the corridor with a maximum width of 400 m were extracted from the results of the previous analysis and superimposed on the divided functional zones. The final ecological function zones were obtained by superimposing them on the functional zones already delineated (Figure 12). Different measures should be developed to enhance ecological maintenance and management according to the characteristics and objectives of each ecological function zone.

According to the zoning results, the ETZs occupy the largest proportion, accounting for 4204% of the total area of the study area. In contrast, the EBZs account for the most minor proportion of the whole area, 13.25%. The ECZs occupy 21.28% of the total area of the study area, and all ecological sources are included. The zones slightly increase in area and connectivity compared to the ecological sources and serve as a protective barrier for ecological sources, a potential area for the expansion and succession of ecological sources. The zones are the core area of ecological protection, development and occupation should be strictly restricted, conservation should be the focus, a reasonable ecological layout should be adopted, natural grassland, ecological woods and other ecological resources restoration projects should be actively implemented (e.g., the “greener mountains” strategic decision implemented by Enshi Prefecture, the various projects around the ecology of the Yangtze River implemented by Yichang City), and ecological protection of various types of cultured forests should be established. For the ECZs with urban construction land areas, the existing parks and cultural scenic spots can be combined to construct and protect urban green corridors and strengthen green space planning to improve the public green space and ecological functions of the urban ECZs. The EBZs are distributed close to ecological sources and are an extension of ecological sources. In the process of the ECZs playing its ecological service function, the EBZs provide enough buffer area to ensure the normal operation of the ECZs and reduce the interference and impact of external human activities on the ECZs. The zones should maintain the dominant landscape stability and focus on optimizing the existing layout to mitigate various land use conflicts. The ETZs are located between the EBZs and EAZs with the largest scale, and the area distribution of cultivated land and construction land in the region is evident. It is a region with prominent contradiction between agricultural production, urban construction and ecological protection. The development and utilization of various kinds of land need to consider the balance between the three and carry out urban construction, tourism development and infrastructure expansion activities appropriately and selectively. Agricultural production should be carried out within reasonable limits, cultivated land should be improved, and the use of chemical fertilizers and pesticides and the discharge of wastewater and waste should be controlled to ensure soil ecological safety. The EAZs account for 23.07% of the total area of the study area and are located outside the ETZs, far away from the ecological sources, and are the zones where urbanization construction and agricultural development are prioritized. The zones should actively expand modernized specialty agriculture based on not affecting or destroying the ecological environment, such as the characteristic citrus, navel orange and tea in Yiling District and Zhijiang City, and white grapefruit in Xuan’en County, etc. These counties can focus on particular products to carry out industrial transformation and upgrading and continuously expand agricultural development. Countries with an excellent rustic foundation can comprehensively promote the large-scale agricultural operation, mechanized production and smart agriculture. When overlaying the ETZs and EAZs layers and layers of cultivated land and construction land, it is found that 74.49% of cultivated land and 84.30% of construction land are within these two zones, indicating that zoning results are more realistic and reliable with the real situation, and they have certain guidance. Within the CRZs, the corridors will be subject to frequent human interference during construction and maintenance, which can be maintained by setting up isolation zones and other measures to strictly control the uncontrolled growth of construction land within the corridors.

### 4.2. Methodological Advantages and Limitations

Currently, the construction and maintenance of ecological networks are essential to ensure the structural integrity of ecosystems in the region and to promote the cyclic flow of ecological streams. The basis of constructing an ecological network is to select suitable ecological sources [72,80]. There are various methods for identifying ecological sources for different conservation objectives and development priorities. Most previous studies have focused only on assessing the supply capacity of ecosystem services or only using the MSPA method alone, with integrated approaches still to be discovered [81]. Compared with other studies that also used an integrated approach to identify ecological sources [28,43,46,49], this study not only proposed a new method to integrate the identification of ecological sources, but also innovated in the subsequent quantification of the importance of each component of ecological network. Firstly, this study demonstrated that the ecosystem service hotspot analysis and MSPA method can effectively help to screen ecological sources in the first stage, and the minimum area threshold and landscape connectivity indices in the later stage can further make the ecological source identification results more accurate. The identified ecological sources provide a good supply of ecosystem services and are critical patches with internal homogeneity and the ability to spread to the surrounding area. Secondly, compared to the traditional method of distinguishing the importance of corridors, this study did not rely on the attractiveness between sources (e.g., only using gravity model) in grading, but considered the functional importance of ecological corridors and the conditions of the corridors themselves, which can provide a certain scientific basis for the priority of corridor protection and restoration. Finally, in this study, the spatial boundaries of the ecological functional zones delineated in the subsequent optimization are clear and in good agreement with the actual situation, providing an important basis for the implementation of ecological spatial protection in mountainous areas.

The study has two limitations. Firstly, this study only identified the ecosystem service capacity of the study area in terms of habitat quality and soil conservation, which was an empirical judgment based on the actual situation of the study area. The combined effects of other ecosystem service functions (e.g., carbon storage, water production) still need to be tested and improved. Secondly, this study did not construct an ecological network based on the living characteristics of specific species in the region, and subsequent studies could be improved to deepen the scientific validity of the results.

## 5. Conclusions

The construction of an ecological network is conducive to realizing the goal of co-development of regional ecological protection and regional development. In this study, taking the MASHP, a typical mountainous area in central China, as the study area, an integrated approach was developed to identify ecological sources based on a quantitative assessment of ecosystem services and MSPA method; an ecological resistance surface that met the characteristics of the regional environment was constructed by considering natural environmental and socioeconomic factors; ecological corridors were extracted according to the least-cost path using the Linkage Mapper tool, and ecological nodes were identified by applying the principle of hydrological analysis; finally, ecological sources, ecological corridors and ecological nodes with different levels of importance together formed the final ecological network in the MASHP, and corresponding optimization measures were proposed.

The main conclusions of this study can be drawn as follows:

(1) This study proposed a new integrated method for identifying ecological sources, considering ecosystem and landscape patch functions and innovates in quantifying the importance of ecological corridors and ecological nodes. The results show that the methods used are feasible and practical.
(2) A total of 49 ecological sources were identified, with an area of 3837.92 km^2^, accounting for 8.51% of the total area in the MASHP. Most of the ecological sources are concentrated in the central region, the county boundary zones in the north and south, and scattered in the southwest, with forestland as the main land use type. A total of 125 ecological corridors were constructed, with a total length of about 2014.61 km. The overall network is connected along the northeast–southwest direction and ran through the whole area, with a dense distribution in the central part. A total of 46 ecological nodes were identified. These nodes are more evenly distributed spatially, only on 42 ecological corridors in total. Ecological sources, ecological corridors and ecological nodes were graded with different levels of importance, ultimately forming a sustainable, composite, multi-level and interconnected ecological network.
(3) Combining the spatial distribution characteristics of each element of the ecological network, the overall layout of ecological network spatial structure is “two verticals, three belts, three groups, and multiple nodes”. To further improve and optimize ecological network in the MASHP, the direction of optimization can be focused on enhancing the connectivity of existing ecological sources with low connectivity, clarifying the width range of ecological corridors and delineating ecological functional zones. The multiple ring buffer analysis was used to optimize the connectivity of ecological sources and improve the effective connection between sources; the optimal range for corridor construction width is 100–400 m, based on local species and landscape structure; the study area was divided into the ecological conservation zones, ecological buffer zones, ecological transition zones, ecological available zones based on ecological sources and minimum cumulative resistance surface. Finally, the corridor restoration zones were added to form the ultimate division result.
(4) Based on the study results, according to the level, spatial structure, distribution characteristics of the components of the ecological network in mountainous areas and the different development positioning of the divided ecological functional zones, this study put forward the recommendations for ecological protection and development and construction planning in mountainous areas. In brief, each ecological source group should focus on forestland protection and water conservation and actively implement ecological resources restoration projects such as natural grassland and ecological forests (e.g., the “greener mountains” strategic decision implemented by Enshi Prefecture, the various projects around the ecology of the Yangtze River implemented by Yichang City), and control anthropogenic activities and construction land boundaries so that each ecological group can better perform its ecological service functions. Ecological corridors are mainly to maintain the ecological stability of the regional microenvironment. Managers may consider setting up isolation zones around ecological corridors of longer lengths (e.g., multiple horizontal axis corridors in the southwest) and setting up buffers by building and protecting artificial green spaces to safeguard the integrity of the ecological network. Meanwhile, different protection measures should be implemented for corridors passing through water and traffic land according to local conditions. It is necessary to strengthen the ecological function of the zone where the ecological nodes are located so that the nodes are less disturbed. On the one hand, it is important to strengthen the nodes within the area of the high original ecological function of green space and water patches and optimization of the plaque. On the other hand, “stepping stone” construction can be carried out through afforestation and ecological park building to improve the connectivity of the overall ecological corridor.

## Figures and Tables

**Table 1:** Data information table.

Data Types	Format	Data Sources
Land use data	Grids at 30 m resolution in 2020	Resource and Environment Science and Data Center (https://www.resdc.c, (accessed on 28 December 2021))
Digital elevation model (DEM)	Grids at 30 m resolution	Geospatial Data Cloud site (http://www.gscloud.cn (accessed on 30 March 2021))
Traffic road and river data	Lines in 2020	National Catalogue Service for Geographic Information (https://www.webmap.cn); OpenStreetMap (https://www.openstreetmap.org (accessed on 30 March 2022))
Meteorological data	Grids at 1 km resolution in 2020	National Meteorological Information Center (https://data.cma.cn/ (accessed on 30 March 2022))
Soil attributes	Grids at 1 km resolution	Harmonized World Soil Database v 1.2 from Food and Agriculture Organization of the United Nations (https://www.fao.org/soils-portal/soil-survey/soil-maps-and-databases/harmonized-world-soil-database-v12/en (accessed on 30 March 2022))
Normalized difference vegetation index (NDVI)	Grids at 1 km resolution in 2019	Resource and Environment Science and Data Center (https://www.resdc.cn (accessed on 30 March 2022))

**Table 2:** Weights and coefficients of resistance factors.

Resistance Factor	Resistance Coefficient					Weight
	10	30	50	70	90	
Land use type	Forestland/Grassland	Water bodies	Cultivated land	Unused land	Construction land	0.5299
Slope (°)	<8	[8–20)	[20–30)	[30–40)	≥40	0.0636
Elevation (m)	<374	[374–755)	[755–1085)	[1085–1441)	≥1441	0.0636
Distance from river (km)	<2	[2–5)	[5–8)	[8–10)	≥10	0.1034
Distance from settlements (km)	≥25	[15–25)	[9–15)	[4–9)	<4	0.1273
Distance from main roads (km)	≥75	[55–75)	[35–55)	[15–35)	<15	0.1122

**Table 3:** Evaluation index system of ecological corridor importance.

Target Layer	Criteria Layer	Solution Layer	Grading Evaluation	Description
Ecological corridor importance	Corridor function importance (0.62)	Total area of patches connected by corridors (0.22)	5	The sum of the area of connected ecological sources by the corridor, the larger the area, the more important the corridor.
			3	
			1	
		Landscape connectivity of main connected ecological sources (0.40)	5	The higher the *dI* of main connected ecological source, the more important the corridor.
			3	
			1	
	Corridor condition (0.38)	Corridor length (0.10)	5	The longer the corridor, the greater the risk of breakage.
			3	
			1	
		Corridor quality I (0.10)	5	The ratio of CWD to the Euclidean distance represents the ease of animal migration between sources. When the value is larger, the corridor quality is poorer
			3	
			1	
		Corridor quality II (0.18)	5	The ratio of CWD to the least-cost path length (LCPL) can study the quality of corridors. When the CWD/LCPL value is larger, species suffer greater resistance to migration or dispersal through this corridor, and the corridor quality is poorer.
			3	
			1	

**Table 4:** Classification criteria of ecological nodes.

Levels of Ecological Nodes	Classification Criteria
1	Located at the intersection of key ecological corridors and the “ridge lines”.
2	Located at the intersection of important ecological corridors and the “ridge lines”.
3	Located at the intersection of ordinary ecological corridors and the “ridge lines”.

**Table 5:** Area of different land use types in corridors with different widths (km^2^).

Land Use Type	Corridor Width (m)						
	30	60	100	400	600	800	1000
Cultivated land	2.49	6.15	15.55	126.66	213.37	295.95	383.74
Forestland	96.85	175.34	311.20	1161.49	1734.31	2305.15	2900.60
Grassland	7.89	14.44	25.42	97.13	145.43	194.68	247.85
Water bodies	1.38	2.76	5.37	25.46	38.69	51.49	64.21
Construction land	0.17	0.39	0.96	6.44	10.97	15.75	21.04

## Data Availability

Not applicable.

## Supplementary Materials

The following supporting information can be downloaded at: https://www.mdpi.com/article/10.3390/ijerph19159582/s1, Table S1: Land use classification system in the study area; Table S2: Threat factors and their stress intensity; Table S3: Habitat suitability and its relative sensitivity to different threat sources; Figure S1: Alternative ecological source patches based on ecosystem service assessment, hotspots analysis and area screening; Figure S2: Alternative ecological source patches based on MSPA analysis and area screening.; Figure S3: Numbered ecological sources in the study area; Table S4: Optimal diffusion distances; Figure S4: Change curve of the rate of forestland increment with the increase in ecological sources buffer distance in the study area; Figure S5: Spatial distribution of optimized ecological sources; Table S5: Classification based on the standard deviation of minimum cumulative resistance.
