# Supplementary material for: Construction and Optimization Strategy of an Ecological Network in Mountainous Areas: A Case Study in Southwestern Hubei Province, China
Source: Int J Environ Res Public Health. 2022 Aug 4;19(15):9582. doi: 10.3390/ijerph19159582 (PMC9368242; doi:10.3390/ijerph19159582)
Supplement: Supplementary file 1 [file ijerph-19-09582-s001.zip › ijerph-1826221-supplementary.pdf]

Supplementary materials for "Construction and optimization strategy of ecological network in mountainous areas: A case study in southwestern Hubei Province, China"

Table S1. Land use classification system in the study area.

**Type**		**Description**
**First class**	**Second class**	
Cultivated land	Paddy field	Cultivated land with guaranteed water source and irrigation facilities, which can be irrigated normally in normal years and used for growing rice, lotus root and other aquatic crops, including cultivated land where rice and dryland crops are rotated.
	Dry land	Cultivated land without irrigation water source and facilities, which grows crops by natural water; dry crop arable land with water source and watering facilities, which can be irrigated normally in a normal year; cultivated land mainly for growing vegetables; recreational land and rotational rest land with normal crop rotation.
Forestland	Woodland	Natural forests and plantations with a denseness of >30%.
	Bush forest	Dwarf and scrub forestland with >40% depression and less than 2 m in height.
	Spare woodland	Forestland with 10-30% tree densities.
	Other woodland	Unestablished afforestation land, trails, nurseries and various types of gardens.
Grassland	High grass cover	Natural grassland, improved grassland and mowed land with >50% cover.
	Medium grass cover	Natural and improved grasslands with >20-50% cover.
	Low grass cover	Natural grassland with 5-20% cover.
Water bodies	Canal	Naturally formed or artificially excavated rivers and land below the perennial water level of the main stem.
	Lake	Land below the perennial water level in naturally occurring waterlogged areas.
	Reservoir pond	Land below the perennial water level in the artificially constructed water storage area.

	Bottom land	Land between the level of the river and lake waters during the flat water period and the level of the flood period.
Construction land	Urban land	Land in large, medium and small cities and construction areas above the county town.
	Rural residential land	Rural settlements that are independent of towns and cities.
	Other construction land	Land for factories, mines, large industrial areas, oil fields, salt fields, quarries, etc., as well as traffic roads, airports and special land.
Unused land	Marshland	Land is flat and low-lying, poorly drained, chronically wet, seasonally waterlogged or perennially waterlogged, with wet plants growing on the surface.
	Bare land	Land with surface soil cover and less than 5% vegetation cover.
	Bare rocky land	Land with a rocky or gravelly surface that covers >5% of the area.

Source: https://www.resdc.cn/data.aspx?DATAID=335

Table S2. Threat factors and their stress intensity.

Threat factor	Maximum impact distance (km)	Weight	Types of spatial decay
Paddy field	5	0.5	exponential
Dry land	5	0.5	exponential
Urban land	10	1	exponential
Rural residential land	5	0.6	exponential
Other construction land	3	0.8	exponential
Main railways	4	0.4	linear
Main roads	3	0.4	linear

Table S3. Habitat suitability and its relative sensitivity to different threat sources.

Land use types	Habitat suitability	Threat factor						
		**Paddy field**	**Dry land**	**Urban land**	**Rural residential land**	**Other construction land**	**Main railways**	**Main roads**
Paddy field	0.50	0	1	0.50	0.70	0.60	0.10	0.20
Dry land	0.40	1	0	0.50	0.70	0.60	0.10	0.20
Woodland	1	0.50	0.60	0.80	0.80	0.80	0.60	0.80
Bush forest	0.80	0.30	0.40	0.60	0.70	0.70	0.60	0.70
Spare woodland	0.75	0.50	0.60	0.90	0.80	0.80	0.50	0.60
Other woodland	0.65	0.50	0.60	0.90	0.80	0.80	0.40	0.50
High grass cover	0.80	0.40	0.45	0.60	0.60	0.60	0.10	0.15
Medium grass cover	0.75	0.45	0.50	0.65	0.70	0.65	0.15	0.20
Low grass cover	0.65	0.50	0.55	0.65	0.70	0.65	0.20	0.25
Canal	1	0.50	0.60	0.85	0.70	0.75	0.40	0.45
Lake	0.90	0.55	0.65	0.90	0.75	0.75	0.45	0.50
Reservoir pond	0.90	0.60	0.70	0.90	0.80	0.80	0.50	0.55
Bottom land	0.60	0.65	0.75	0.80	0.75	0.75	0.55	0.60
Urban land	0	0	0	0	0	0	0	0
Rural residential land	0	0	0	0	0	0	0	0
Other construction land	0	0	0	0	0	0	0	0
Marshland	0.60	0.50	0.60	0.80	0.60	0.50	0.60	0.70
Bare land	0	0	0	0	0	0	0	0
Bare rocky land	0	0	0	0	0	0	0	0

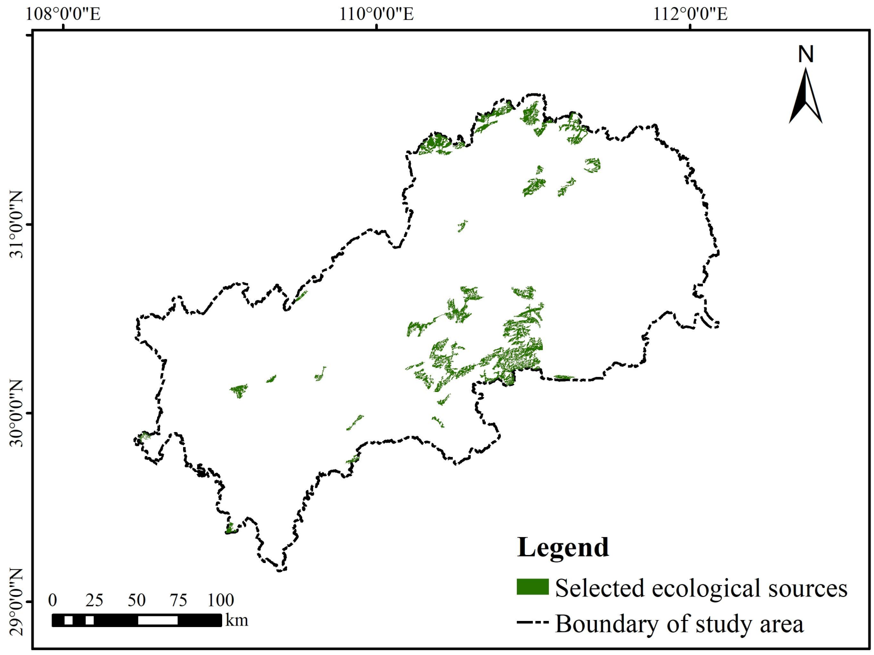

Figure S1. Alternative ecological source patches based on ecosystem service assessment, hotspots analysis and area screening.

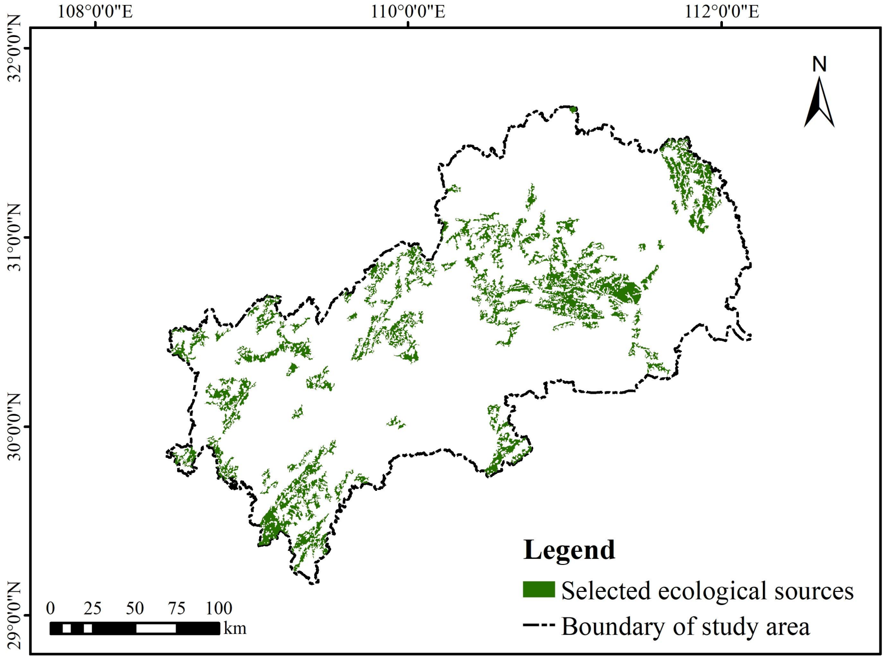

Figure S2. Alternative ecological source patches based on MSPA analysis and area screening.

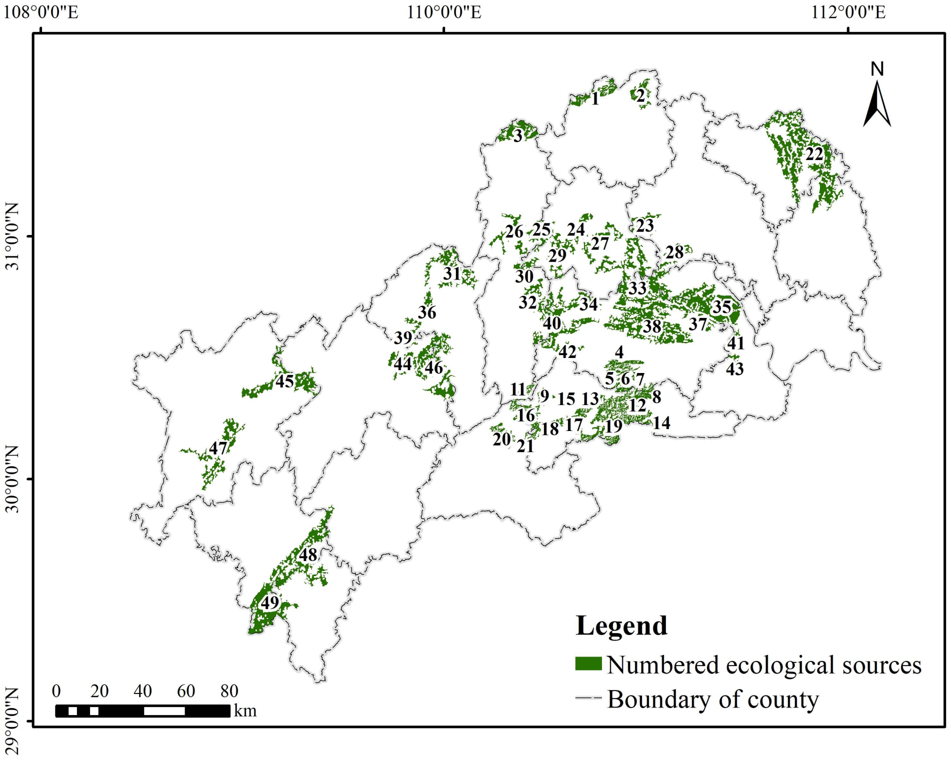

Figure S3. Numbered ecological sources in the study area.

Table S4. Optimal diffusion distances.

Ecological source number	Optimal diffusion distance (m)	Ecological source number	Optimal diffusion distance (m)
1	600	36	100
2	500	37	100
9	400	39	200
10	500	41	100
14	900	42	100
23	400	43	100
28	100	45	100
30	300	47	400
31	200		
32	200		

Note: The number of ecological sources corresponds to Figure S3.

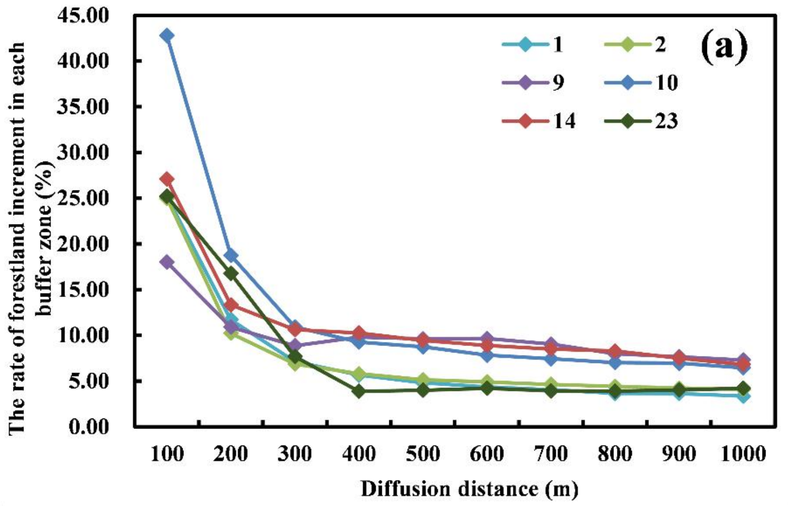

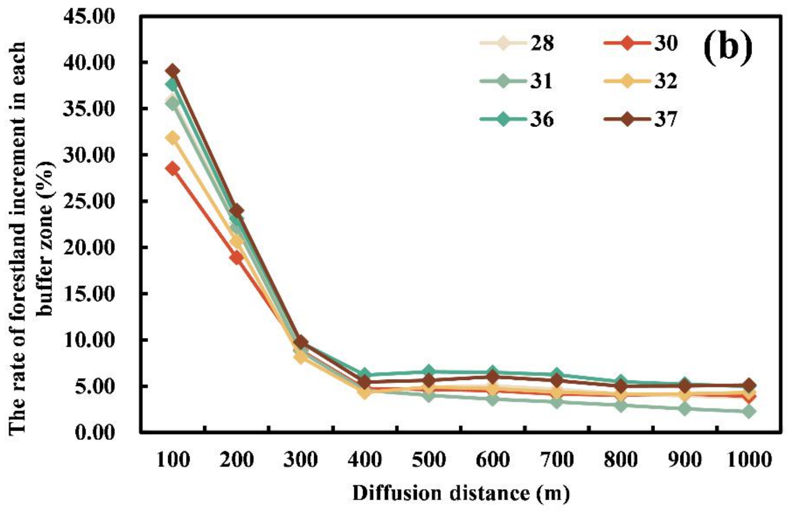

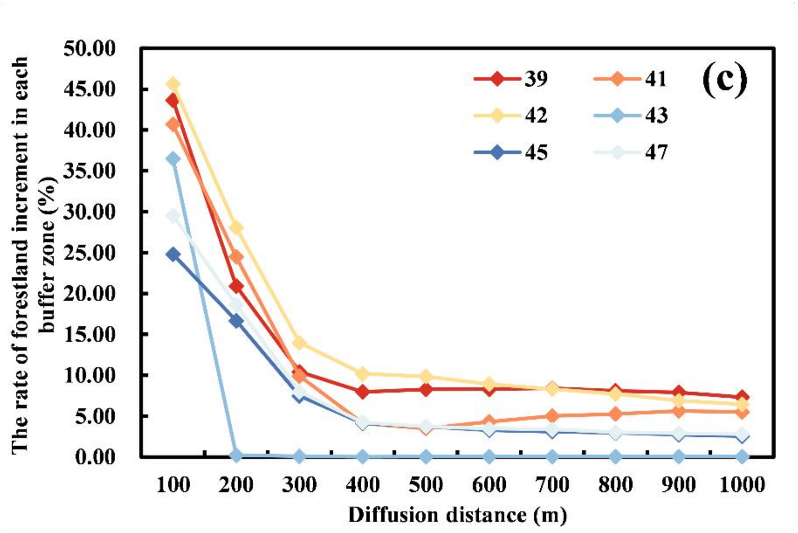

Figure S4. Change curve of the rate of forestland increment with the increase of ecological sources buffer distance in the study area: (a)number 1,2,9,10, 14 and 23; (b)number 28,30.31,32,36 and 37; (c)number 39,41,42,43,45 and 47.

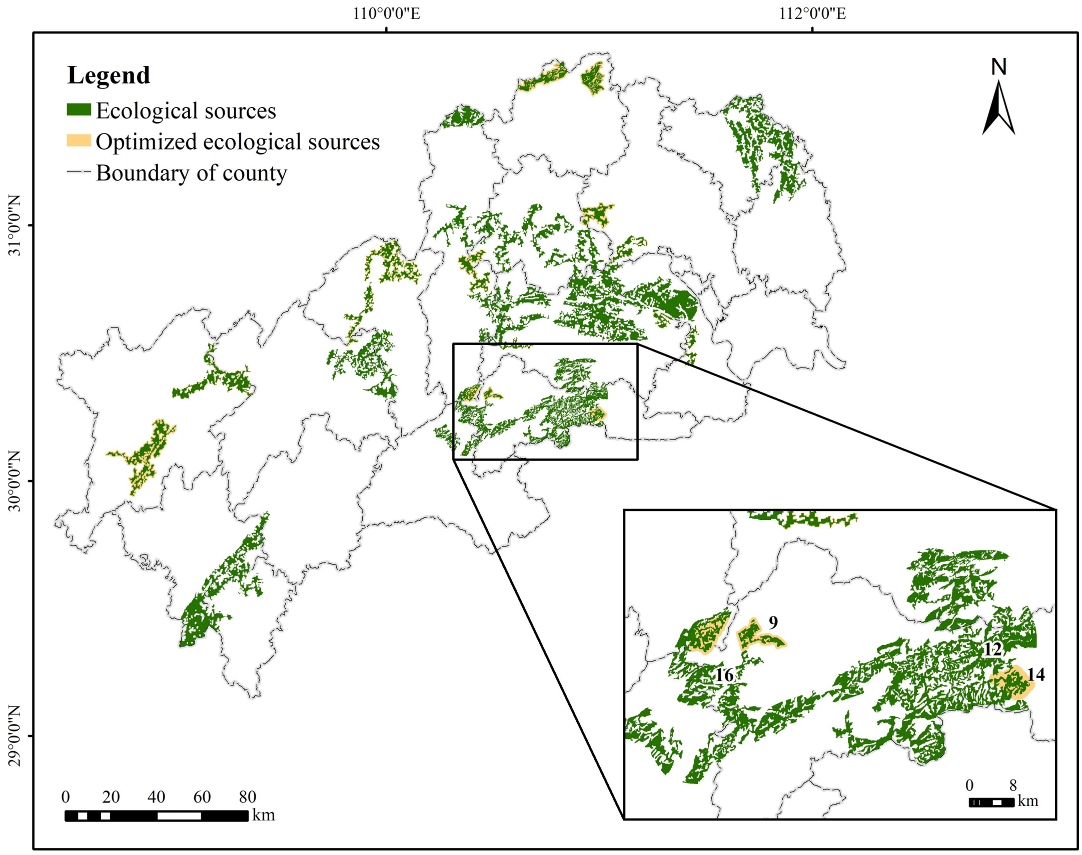

Figure S5. Spatial distribution of optimized ecological sources.

Table S5. Classification based on the standard deviation of minimum cumulative resistance.

Classification	Value range	Grid number	Proportion (%)	Ecological function zone
C1	0—38,183	10,664,658	21.28	Ecological conservation zone
C2	38,184—131,896	6,643,914	13.25	Ecological buffer zone
C3	131,897—225,610	5,114,100	10.20	Ecological transition zone
C4	225,611—319,323	4,521,963	9.02	
C5	319,324—413,036	4,194,532	8.37	
C6	413,037—506,749	3,896,765	7.77	
C7	506,750—600,462	3,521,618	7.03	
C8	600,463—694,175	2,942,096	5.87	Ecological available zone
C9	694,176—787,888	2,371,752	4.73	
C10	787,889—881,601	1,868,419	3.73	
C11	881,602—975,314	1,222,657	2.44	
C12	975,315—1,069,027	847,598	1.69	
C13	1,069,028—1,162,740	610,597	1.22	
C14	1,162,741—1,256,453	386,916	0.77	
C15	1,256,454—1,350,166	232,913	0.46	
C16	1,350,167—1,443,880	161,181	0.32	
C17	1,443,881—2,859,299	922,332	1.84	
Total		50,124,011	100.00	
